# O-GlcNAc regulates the mitochondrial integrated stress response by regulating ATF4

**DOI:** 10.3389/fnagi.2023.1326127

**Published:** 2023-12-18

**Authors:** Ibtihal M. Alghusen, Marisa S. Carman, Heather Wilkins, Sophiya John Ephrame, Amy Qiang, Wagner B. Dias, Halyna Fedosyuk, Aspin R. Denson, Russell H. Swerdlow, Chad Slawson

**Affiliations:** ^1^School of Medicine, Department of Biochemistry and Molecular Biology, University of Kansas Medical Center, Kansas City, KS, United States; ^2^Department of Neurology, University of Kansas Medical Center, Kansas City, KS, United States; ^3^University of Kansas Alzheimer’s Disease Research Center, University of Kansas Medical Center, Kansas City, KS, United States

**Keywords:** O-GlcNAc, mitochondrial stress, integrated stress response, activating transcription factor 4 (ATF4), Alzheimer’s disease

## Abstract

**Background:**

Accumulation of mitochondrial dysfunctional is a hallmark of age-related neurodegeneration including Alzheimer’s disease (AD). Impairment of mitochondrial quality control mechanisms leading to the accumulation of damaged mitochondria and increasing neuronal stress. Therefore, investigating the basic mechanisms of how mitochondrial homeostasis is regulated is essential. Herein, we investigate the role of O-GlcNAcylation, a single sugar post-translational modification, in controlling mitochondrial stress-induced transcription factor Activating Transcription Factor 4 (ATF4). Mitochondrial dysfunction triggers the integrated stress response (ISR^mt^), in which the phosphorylation of eukaryotic translation initiation factor 2α results in the translation of ATF4.

**Methods:**

We used patient-derived induced pluripotent stem cells, a transgenic mouse model of AD, SH-SY5Y neuroblastoma and HeLa cell-lines to examine the effect of sustained *O*-GlcNAcase inhibition by Thiamet-G (TMG) on ISR^mt^ using biochemical analyses.

**Results:**

We show that TMG elevates ATF4 protein levels upon mitochondrial stress in SH-SY5Y neuroblastoma and HeLa cell-lines. An indirect downstream target of ATF4 mitochondrial chaperone glucose-regulated protein 75 (GRP75) is significantly elevated. Interestingly, knock-down of O-GlcNAc transferase (OGT), the enzyme that adds O-GlcNAc, in SH-SY5Y increases ATF4 protein and mRNA expression. Additionally, ATF4 target gene Activating Transcription Factor 5 (ATF5) is significantly elevated at both the protein and mRNA level. Brains isolated from TMG treated mice show elevated levels of ATF4 and GRP75. Importantly, ATF4 occupancy increases at the ATF5 promoter site in brains isolated from TMG treated mice suggesting that O-GlcNAc is regulating ATF4 targeted gene expression. Interestingly, ATF4 and GRP75 are not induced in TMG treated familial Alzheimer’s Disease mice model. The same results are seen in a human *in vitro* model of AD.

**Conclusion:**

Together, these results indicate that in healthy conditions, O-GlcNAc regulates the ISRmt through regulating ATF4, while manipulating O-GlcNAc in AD has no effect on ISR^mt^.

## Introduction

Mitochondrial dysfunction is a common pathological hallmark for neurodegenerative diseases (ND). Mitochondrial abnormalities in ND include alterations in mitochondrial morphology and mitochondrial respiration (Selfridge et al., 2013). In fact, the accumulation of dysfunctional mitochondria is one of the early features of Alzheimer’s disease (AD). However, the alterations in mitochondrial homeostasis driving this accumulation are still unknown. The impairment of various mitochondrial retrograde signaling (mitochondrial-to-nuclear signaling) pathways contributes to AD progression. In normal conditions, retrograde signaling is activated upon mitochondrial stress, leading to changes in nuclear gene expression, which facilitate mitochondrial recovery. For example, one mitochondrial-to-nuclear pathway that controls mitochondrial homeostasis is the mitochondrial integrated stress response (ISR^mt^). The ISR^mt^ regulates the transcriptional response of mitochondrial chaperones in response to mitochondrial stress, including perturbed protein import arising from protein misfolding, the disruption of mitochondrial inner membrane potential, and ATP depletion (Nargund et al., 2012). Aberrant ISR^mt^ signaling is associated with many age-related diseases, including AD characterized by the accumulation of unfolded and aggregated proteins affecting the nervous system (Sorrentino et al., 2017). The mRNA expression of mitochondrial stress response related genes found at significantly higher level in patients with mild and moderate AD compared to normal individuals (Sorrentino et al., 2017).

**Figure 1:**
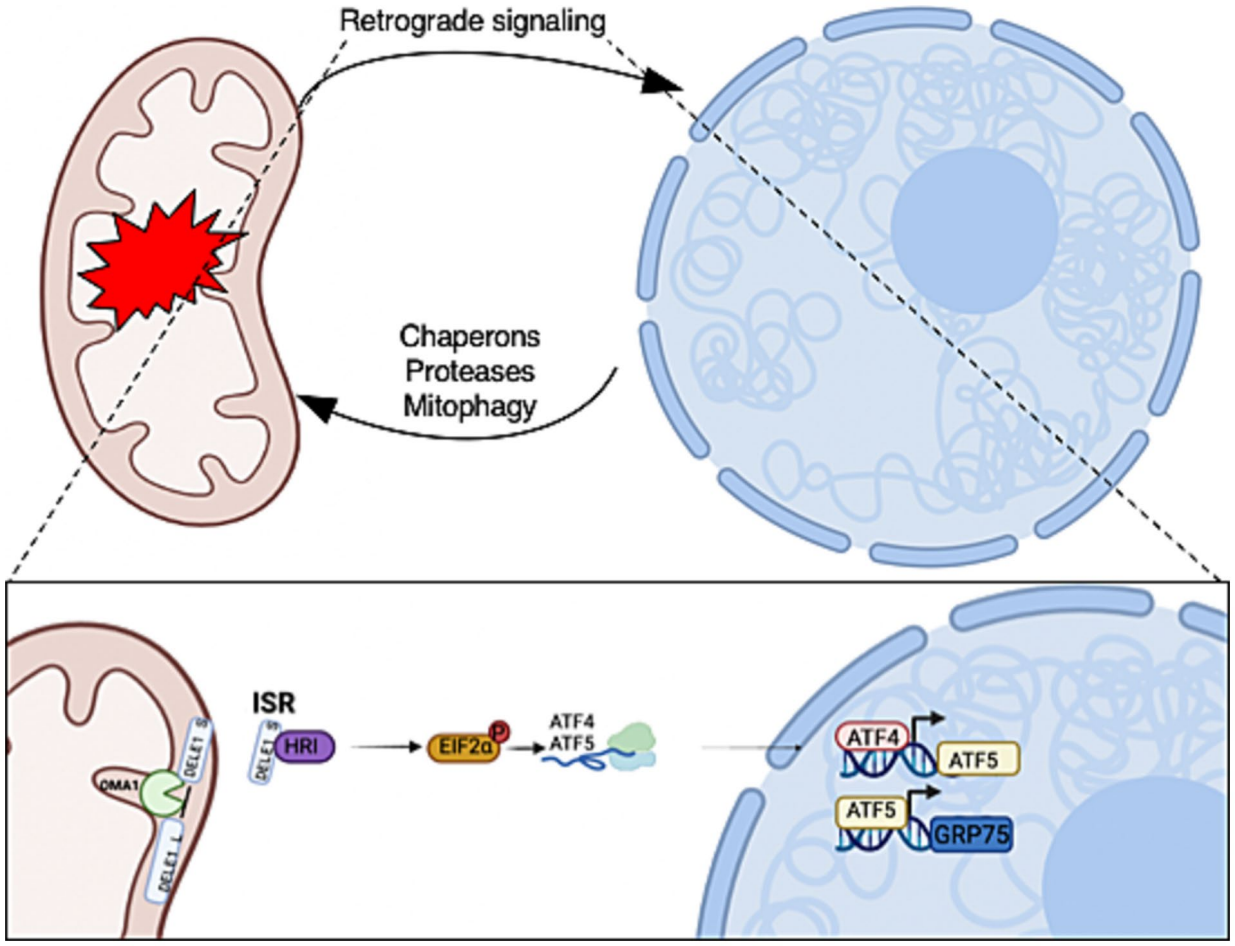
Mitochondrial-to-nuclear communication is triggered in response to mitochondrial stress. The retrograde signals allow mitochondria to communicate stress conditions to regulate the expression of nuclear-encoded genes for adaptations. The activation of retrograde transcription factors increases the expression of mitochondrial chaperons, proteases, and mitophagy genes that are required for mitochondrial recovery and homeostasis. Mitochondrial stress initiates OMA1 zinc metallopeptidase (OMA1) to cleave DAP3 binding cell death enhancer 1 (DELE1) on the inner mitochondrial membrane. A fragment of DELE1 enters the cytosol and binds to heme-regulated eIF2α kinase (HRI), which activates it. The HRI phosphorylate eukaryotic Initiation Factor 2 (P-eIF2α) increases the translation of ATF4 and ATF5. These transcription factors are required to increase the expression of nuclear stress-response genes to initiate the mitochondrial integrated stress response (ISR^mt^). Created with BioRender.com.

Numerous mitochondrial stressors trigger the integrated stress response, leading to the phosphorylation of eukaryotic translation initiation factor 2α (eIF2α) and resulting in the translation of the transcription factors activating transcription factor 4 (ATF4) and activating transcription factor 5 (ATF5) (Weidling and Swerdlow, 2019). ATF4 initiates the expression of ATF5 (Teske et al., 2013; Juliana et al., 2017), which in turn increases the gene expression of mitochondrial chaperone glucose-regulated protein 75 (GRP75), a member of heat shock protein family A (Figure 1). Interestingly, ATF4 is found at high levels in AD, but cells fail to restore mitochondrial function. Neurons are heavily O-GlcNAcylated (Cole and Hart, 2001; Wulff-Fuentes et al., 2021), and aberrant O-GlcNAcylation is observed in the AD brain (Griffith and Schmitz, 1995; Liu et al., 2009; Park et al., 2021). Several studies demonstrated that O-GlcNAc mitigates neurodegeneration (Yuzwa et al., 2012; Hastings et al., 2017; Park et al., 2021); therefore, we hypothesize that the elevation of ATF4 in AD is associated with disruptions in O-GlcNAcylation.

O-GlcNAc is a ubiquitous post-translational modification involving the attachment of a single N-acetylglucosamine moiety to serine or threonine residues on nuclear, cytoplasmic, and mitochondrial proteins. The dynamic addition and removal of the sugar by O-GlcNAc transferase (OGT) or O-GlcNAcase (OGA), respectively, maintains a homeostatic level of cellular O-GlcNAc. O-GlcNAcylation regulates mitochondrial function (Sacoman et al., 2017; Tan et al., 2017; Dontaine et al., 2022). Overexpression of either OGT or OGA in SH-SY5Y neuroblastoma cells lowers mitochondrial respiration, disrupts morphology, and decreases the expression of respiratory chain and tricarboxylic acid cycle (TCA) proteins (Tan et al., 2014). Numerous mitochondrial proteins are modified by O-GlcNAc (Ma et al., 2016), and both OGT and OGA localize to the mitochondria (Banerjee et al., 2015). Acute OGT and OGA inhibition significantly affects mitochondrial respiration, ATP production, and membrane potential (Banerjee et al., 2015). Furthermore, the inhibition of OGA lowers electron transport chain activity, increases the NAD+/NADH ratio, and reduces ROS output (Tan et al., 2017). Together, these studies show a critical role for O-GlcNAc in regulating mitochondrial function both directly through the O-GlcNAcylation of mitochondrial proteins and indirectly by controlling metabolic gene expression. However, the role of O-GlcNAc in regulating ISR^mt^ is still unknown in both normal and AD conditions.

Here, we report that initiating mitochondrial stress by inhibiting OGA elevates ATF4 expression in two different cell lines. The elevation of ATF4 by inhibiting OGA is replicated in mice neuronal tissue. Moreover, OGT knockdown (KD) in SH-SY5Y neuroblastoma cells elevates ATF4 and ATF5 mRNA and protein expression. Importantly, we show higher binding of ATF4 at the ATF5 promoter site in mice neuronal tissue after OGA inhibition. However, ATF4 response to O-GlcNAc alteration is impaired in both an AD mouse model and human AD organoid models. Together, these data demonstrate distinct roles for O-GlcNAc in modulating mitochondrial retrograde signaling through ATF4. However, in AD, the failure of retrograde signaling to respond to O-GlcNAc alteration is unknown.

## Materials and methods

### Cell culture

Both SH-SY5Y and HELA cells were cultured in DMEM (Sigma-Aldrich, D5030-10L), supplemented with 4 mM glucose, 15 mg/L phenol red (Sigma-Aldrich), 44 mm sodium bicarbonate (Sigma-Aldrich), 1% GlutaMAX (Gibco), 10% fetal bovine serum (FBS; Gemini), and 1% penicillin/streptomycin (Sigma-Aldrich). Cells were treated with 10 μm thiamet-G (SD Specialty Chemicals) (TMG, from a 20 mM stock in Tris-Buffered Saline [pH 7.4]) for at least 2 weeks before the experiments. The medium was replenished daily. Cells were harvested for total lysate at the different time points 1, 2, 4, 6, and 8 h upon mito-stress stimulation (UA).

### Lentivirus preparation

The OGT knockdown (KD) and OGA KD cells were generated by sh-RNA mediated lentiviral KD (ThermoFisher). Plasmids of OGT KD shRNAs, OGA KD shRNAs, and scramble GFP shRNA, along with plasmids that encode for lentiviral particles, were purified using a maxiprep kit (Sigma-Aldrich, NA0310). HEK293T cells were seeded at a density of 5 × (10^6) cells in each 10 cm dish with a total volume of 10 mL DMEM (25 mM Glucose) during day 1. During day 2, each HEK293T cell-plate was transfected using the TransIT-X2^®^ Dynamic Delivery System (Mirus MIR 6005) in 1.5 mL of opti-MEM serum-free medium (Thermo Fisher, 11058021) by adding OGT or OGA shRNA plasmids along with PCMV and PMD2G plasmids encoding the viral coat. The conditioned medium containing lentivirus was collected the next day and stored at −20°C. The process was repeated for 2 additional days. Finally, the conditioned medium was centrifuged at 1,000 *g* for 3 min and the supernatant was passed through a 0.45 μm filter to make lentivirus infection medium.

### shRNA lentivirus infection

SH-SY5Y cells were plated in 10-cm dishes–one for scramble and one for each target shRNA. The next day, the culture medium was discarded in each plate and replaced with 4 mL offresh medium and 4 mL lentivirus for infection. The culture medium in each plate was discarded the next day and replaced with 10 mL of fresh medium supplied with puromycin at 1 μg/mL. The process was continued for the next 4–9 days.

### Animal protocols and models

The University of Kansas Medical Center Animal Care and Use Committee approved all experiments in this study. Two-month-old male C57Bl/6J mice were purchased from The Jackson Laboratory (Bar Harbor, ME, USA). All mice were housed using a standard 12-h light/dark cycle with access to chow and water *ad libitum*. Mice were treated with a 50 mg/kg thiamet-G intraparietal injection every other morning for 30 days or 6 months. After the completion of dosing, mice were fasted for 16 h before isoflurane (Fisher) anesthesia assisted cervical dislocation.

### Cell lysis and immunoblotting

Cells were lysed on ice in Np-40 lysis buffer (containing 20 mM Tris [pH 7.4], 150 mM NaCl, 40 mM GlcNAc, 2 mM EDTA, 1 mM DTT, and 1% Nonidet P-40 with the phosphatase inhibitors β-glycerophosphate [1 mM] and Sodium Fluoride [NaF, 1 mM], and the protease inhibitors phenylmethylsulfonyl fluoride [PMSF, 2 mM] and a 1 × inhibitor mixture composed of 1 μg/mL leupeptin, 1 μg/mL antipain, 10 μg/mL benzamidine, and 0.1% aprotinin added immediately before lysis). Animal tissues were lysed with RIPA buffer (containing 10 mM Tris [pH 7.6], 150 mM NaCl, 40 mM GlcNAc, 2 mM EDTA, 1 mM DTT, 1% Nonidet P-40, 0.1% SDS, 0.5% deoxycholic acid with the phosphatase inhibitors β-glycerophosphate (1 mM), 1 mM Sodium Fluoride (NaF, 1 mM), and protease inhibitors 2 mM phenylmethylsulfonyl fluoride (PMSF, 2 mM) and 1 × inhibitor mixture composed of 1 μg/mL leupeptin, 1 μg/mL antipain, 10 μg/mL benzamidine, and 0.1% aprotinin added immediately before lysis). All of the above components were purchased from Sigma-Aldrich. Lysates were incubated on ice for 20 min and vortexed every 5 min. The protein concentration of the lysate was determined using a Bradford (Bio-Rad Catalog) or BCA assays (Thermo Fisher). Lysates were then denatured through the addition of a 4 × protein solubility mixture (100 mM Tris [pH 6.8], 10 mM EDTA, 8% SDS, 50% sucrose, 5% β-mercaptoethanol, and 0.08% Pyronin Y) and boiling for 2 min. Equal protein amounts of lysates were loaded onto 4–15% Criterion precast TGX gels (Bio-Rad). Electrophoresis was carried out at 130V for approximately 50 min and then the gel proteins were transferred to polyvinylidene difluoride (PVDF) membranes at 0.4 A. Membranes were blocked with 3% BSA and 0.01% sodium azide in TBST (25 mM Tris [pH 7.6], 150 mM NaCl, and 0.05% Tween 20) for at least 20 min. Blots were then probed overnight at 4°C with primary antibody against the protein of interest at a dilution of 1:1,000. The next day, the blots were washed three times in TBST for 10 min each time. HRP-conjugated secondary antibody (Bio-Rad) at a dilution of 1:10,000 was added for 1 h at room temperature, followed by washing three times for 10 min each time. Blots were then developed using the chemiluminescence HRP antibody detection method (Thermo Fisher, 34095). The blots were striped with 200 mM glycine [pH 2.5] for 1 h at room temperature, blocked, and re-probed. Where shown, ImageJ 3.2 (National Institutes of Health) or image Lab (Bio-Rad) were used to quantify the density of the protein bands compared with an internal standard protein band, such as GAPDH or α-Tubulin. At least three independent experiments were repeated for all immunoblotting.

### Antibodies

Primary and secondary antibodies for immunoblotting were used at dilutions of 1:2,000 and 1:10,000, respectively. OGT (AL-34), and OGA (345) were gracious gifts from the laboratory of Gerald Hart in the Department of Biochemistry and Molecular Biology at the University of Georgia. The other antibodies used were as follows: anti-*O*-linked *N*-acetylglucosamine antibody (RL2, Thermo Fisher, MA1-072), anti-GAPDH antibody (Abcam, ab9484), GRP75 (Cell Signaling Technology, D13H4), ATF5 (Abcam, ab184923), ATF4 (Cell Signaling Technology, D4B8), TOM20 (Santa Cruz Biotechnology, F-10, sc-17764), α-tubulin (Sigma-Aldrich, T5168), anti-chicken IgY-HRP (Sigma-Aldrich, A9046), goat anti-rabbit IgG-HRP (Bio-Rad, 170–6515), and goat anti-mouse IgG-HRP (Bio-Rad, 170–6516).

### Mitochondrial purification from cells and brains

Mitochondria were isolated from SH-SY5Y and HELA cells using the cavitation method. Briefly, 2 × 10^8^ cells were trypsin digested off the plate and washed twice with pre-chilled PBS and then resuspended in 3 mL of the mitochondrial isolation medium. The cell suspensions were transferred into a pre-chilled cavitation chamber (nitrogen bomb; Parr Instrument Co., Moline, IL, USA) and subjected to 900 p.s.i. for 15 min. Subsequently, the pressurized cell suspension was collected from the cavitation chamber, followed by centrifugation at 1,000 × *g* for 10 min to pellet the cell debris. The clear supernatant was collected and centrifuged at 12,000 × *g* for 15 min. The crude mitochondrial pellet was washed three times with 500 μL of isolation medium. The washed pellet was lysed with Nonidet P-40 lysis buffer.

Mouse brain mitochondria were isolated from the whole brain using the Percoll gradient and ultracentrifugation. All reagents were pre-chilled on ice. The brain was rinsed twice with ice-cold PBS, then scissored and homogenized (Teflon glass homogenizer) in 5 mL of mitochondria isolation buffer (MIB; 225 mM mannitol, 75 mM sucrose, 6 mM K2HPO4, 1 mM EGTA, and 0.1% fatty acid-free BSA, pH 7.2). The resulting homogenate was centrifuged at 1,500 × *g* for 5 min at 4°C. Next, 15, 23, and 40% Percoll gradients were made using 100% Percoll in MIB. The 40% Percoll gradient (2.3 mL) was layered on the bottom of a centrifuge tube, followed by 2.3 mL of 23% Percoll (middle), then 2.3 mL of 15% Percoll (top). The supernatant (5 mL) was added to the top of the layered Percoll gradients. Ultracentrifugation was carried out using an SW28.1 Beckman rotor; the tubes were centrifuged at 7,800 rpm for 15 min at 4°C. The mitochondrial layer was collected and washed with 8 mL of MIB and centrifuged at 8,000 × *g* for 10 min. Mitochondria were then washed a second time with 8 mL of sterile phosphate-buffered saline (PBS) and re-centrifuged at 8,000 × *g* for 10 min. The precipitated mitochondria were resuspended, gently, in 500 μL of MIB.

### Total RNA isolation

Total RNA was isolated from SH-SY5Y and HELA cells (5 × 10^6^) cells using 1 mL of TRI Reagent (Sigma-Aldrich, T9424). The RNA concentration was measured using a Nanodrop 2000c (Thermo Fisher Scientific). For the RT reactions, 0.5 μg of total RNA was reverse transcribed to cDNA using iScript Reverse Transcription Supermix (Bio-Rad, 170–8841). The reactions were incubated in a thermal cycler (Model 2720, Applied Biosystems) using the following protocol: priming for 5 min at 25°C, RT for 20 min at 46°C, RT inactivation for 1 min at 95°C, and hold at 4°C. The cDNA products were diluted with nuclease-free water (1:10 dilution) and analyzed by qPCR.

### RT-qPCR

RT-qPCR was performed on cDNA samples using SsoAdvanced Universal SYBR Green Supermix (Bio-Rad) following the manufacturer’s instructions. The following primers were used: ATF4, F, 5′-CCAAGCACTTCAAACCTCATGG-3′, and R, 5′-GAGAAGGCATCCTCCTTGCTG-3′; ATF5, F, 5′-GCTCGTAGACTATGGGAAACTCC-3′, and R, 5′-CATCCAGTCAGAGAAGCCATCAC-3′; ATF5 promoter, F, 5′-TCCCTCAGCCCTTCCTAACCATC-3′, and R, 5′-AGGGTGGAGCTGCTTGGAACTC-3′.

### qPCR data analysis

For cDNA RT-qPCR data, the dynamic ranges of RT and amplification efficiency were evaluated before applying the ΔΔCq method to calculate relative gene expression change. The transcription level of the target gene was normalized to the internal control as fold change. Data generated in at least three independent experiments are presented as mean ± S.E. A two-tailed Student’s *t* test statistic was applied, with *p* < 0.05 indicating a significant difference.

### Chromatin immunoprecipitation assay (ChIP)

The ChIP assay was performed using an Abcam high-sensitivity ChIP kit (Abcam, ab185913), following the manufacturer’s protocol. qPCR was performed as described previously to calculate the fold enrichment after ChIP. The Ct values of ATF4-IP were normalized to rabbit IgG values.

### Nucleus/cytoplasm fractionation

Preparations were performed on ice. Cells were resuspended in 200 ul hypotonic solution containing 0.1% NP-40 (20 mM HEPES [pH 7.5], 50 mM NaF, 5 mM Na_2_P_2_O_7_, 50 mM GlcNAc, 1 mM EDTA, 1 mM EGTA, 1 mM dithiothreitol [DTT], 1 mM PMSF, and a protease inhibitor cocktail). After pipetting a couple of times, lysates were centrifuged at 2,900 × *g* for 30 s to pellet nuclei and transfer cytoplasmic extract. The nuclei pellet was washed with 500 μl hypotonic solution without 0.1% NP40 four times before resuspension in 40 μL lysis buffer (containing 20 mM Tris [pH 7.4], 150 mM NaCl, 40 mM GlcNAc, 2 mM EDTA, 1 mM DTT, 1% Nonidet P-40 with the phosphatase inhibitors β-glycerophosphate (1 mM) and sodium fluoride (NaF, 1 mM), and the protease inhibitors phenylmethylsulfonyl fluoride (PMSF, 2 mM) and 1 × inhibitor mixture composed of 1 μg/mL leupeptin, 1 μg/mL antipain, 10 μg/mL benzamidine, and 0.1% aprotinin added immediately before lysis). The nuclear extract was then centrifuged at 15,700 × *g* for 20 min at 4°C. The resulting supernatant was collected as total nuclear extract.

### iPSC source and reprogramming

iPSCs were reprogrammed from dura fibroblasts obtained from the University of Kansas Alzheimer’s Disease Research Center (KU ADRC) or purchased from WiCell. KU ADRC fibroblast donors were members of the clinical cohort who consented to donation upon death, and approval from an ethical standards committee to conduct this study was received. The studies involving human participants were reviewed and approved by the University of Kansas Medical Center Institutional Review Board. Banked tissue was de-identified by the KUADRC Neuropathology Core to eliminate identifying information. Reprogramming was completed using the Sendai Virus, CytoTune-iPS 2.0 *Sendai Reprogramming* Kit from Thermo Fisher. The iPSCs were age, sex, and diagnosis matched (Supplementary Table 1). For iPSCs derived from the KU ADRC cohort, ND or AD were diagnosed at the autopsy neuropathological examination, as outlined in the NACC Neuropathology Coding Guidebook ed2020.

### Cerebral organoid generation

Cerebral organoids were made using iPSCs and StemDiff Cerebral Organoid kits from STEMCELL. The iPSCs were briefly placed into single cell suspensions with ROCKi in embryoid body formation plates. Embryoid bodies were expanded for 7 days and then embedded into Matrigel droplets. The organoids were matured for 90 days.

## Results

**Figure 2:**
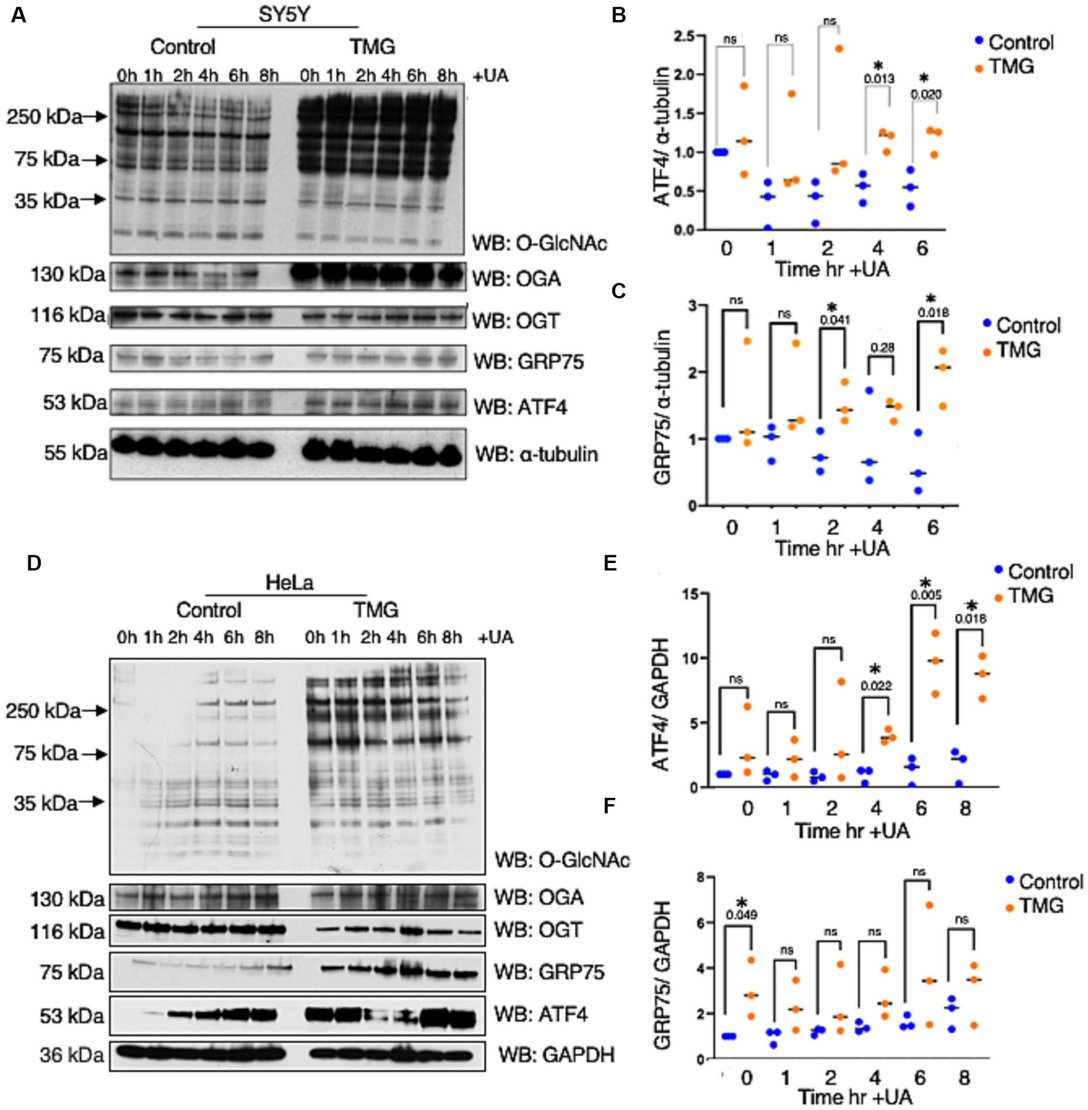
TMG initiates ISR upon mitochondrial stress in human cell lines. Western blot analysis of samples harvested after stimulating mitochondrial stress using a UA time course in SH-SY5Y neuroblastoma cells **(A–C)** and HeLa cervical cancer cells **(D–F)** subjected to long-term OGA inhibition with TMG. **(B,E)** Densitometry plot of ATF4 normalized to a loading control. **(C,F)** Densitometry plot of GRP75 normalized to a loading control. SH-SY5Y cells (*n* = 3) in **(B,C,E,F)** and HeLa (*n* = 3), in which dots represent the number of experimental trials (n). Statistical significance was measured using an unpaired *t*-test analysis, and the *p*-values are indicated on the plots. *p-values that are significant (*p* < 0.05). ns, not significant.

Most studies show increase O-GlcNAc pharmacologically mitigates neurodegeneration focusing on Tau and amyloid precursor protein with limited studies on ISR. Age-related cognitive diseases are associated with higher ISR^mt^ signaling (Moreno et al., 2012; Kim et al., 2014; Chou et al., 2017; Rosi and Frias, 2020). To determine how *O*-GlcNAcylation affects ATF4 and its signaling, we subjected the neuroblastoma cell line (SH-SY5Y) and cervical cancer cells (Hela) to long-term TMG treatment (10 μm) for at least 3 weeks prior to harvesting. Additionally, we treated the cells with Urolithin A (UA, 50 μm) to initiate mitochondrial stress through stimulating mitophagy (Fang et al., 2019). Cells were harvested for total lysate at different time points 1, 2, 4, 6, 8 h after the addition of UA. TMG increased O-GlcNAc in SH-SY5Y and Hela cells as expected (Figures 2A,D). TMG increased OGA expression, and a decline in OGT expression was evident in the total lysate of TMG-treated cells (Figures 2A,D). In both cell lines, ATF4 showed a significant protein elevation in TMG-treated cells 4 and 6 h after UA treatment (Figures 2A,B,D,E). We next measured the protein expression of the downstream target of ISR^mt^ activation, the mitochondrial chaperon GRP75. GRP75 showed a significant elevation in TMG- and UA-treated SH-SY5Y, while in HeLa, GRP75 increased significantly without the need of stimulating mitophagy (Figures 2A,C,D,F).

**Figure 3:**
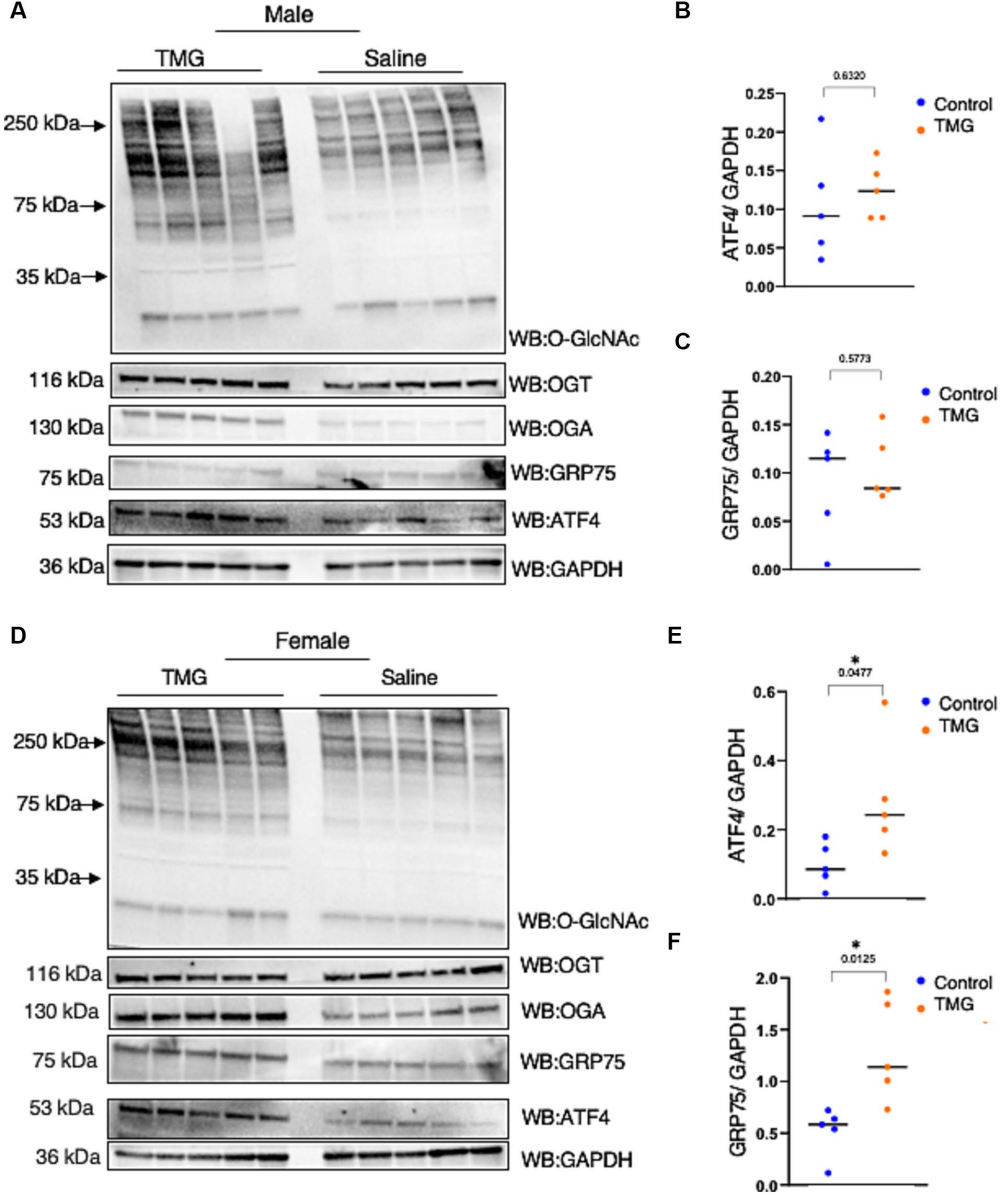
The ISR in TMG-treated mice brains is elevated. **(A)** Western blot analysis of the total brain lysate of WT-C57BL/6J male mice subjected to intraperitoneal TMG injections for 1 month. The blots were probed for O-GlcNAc, OGT, OGA, GAPDH, GRP75, and ATF4 **(A)**. **(B)** Densitometry plot of ATF4 normalized to GAPDH. **(C)** GRP75 normalized to GAPDH. **(D)** Western blot analysis of the total brain lysate of WT-C57BL/6J female mice subjected to intraperitoneal TMG injections for 1 month. The blots were probed for O-GlcNAc, OGT, OGA, GAPDH, GRP75, and ATF4 **(D)**. **(E)** Densitometry plot of ATF4 normalized to GAPDH. **(F)** GRP75 normalized to GAPDH. The dots represent the number of mice (*n* = 5 for the control and *n* = 5 for TMG-injected mice). Statistical significance was measured using an unpaired *t*-test analysis and *p*-values are indicated on the plots. *p-values that are significant (*p* < 0.05).

Because TMG induced ATF4 upon mitochondrial stress in cell lines, we investigated whether the brains of C57BL/6J mice with TMG intraperitoneally injected for 1 month replicated the cell line data. Brains were harvested and homogenized for total lysate and a western blot analysis was carried out. Males TMG treated show a slight increase in ATF4 and a slight decrease in GRP75 compared to saline (Figures 3A–C). TMG-treated males showed a slight increase in ATF4 compared with saline (Figures 3A,B). However, TMG-injected females showed a significant elevation in ATF4 and GRP75 compared with saline (Figures 3D–F).

**Figure 4:**
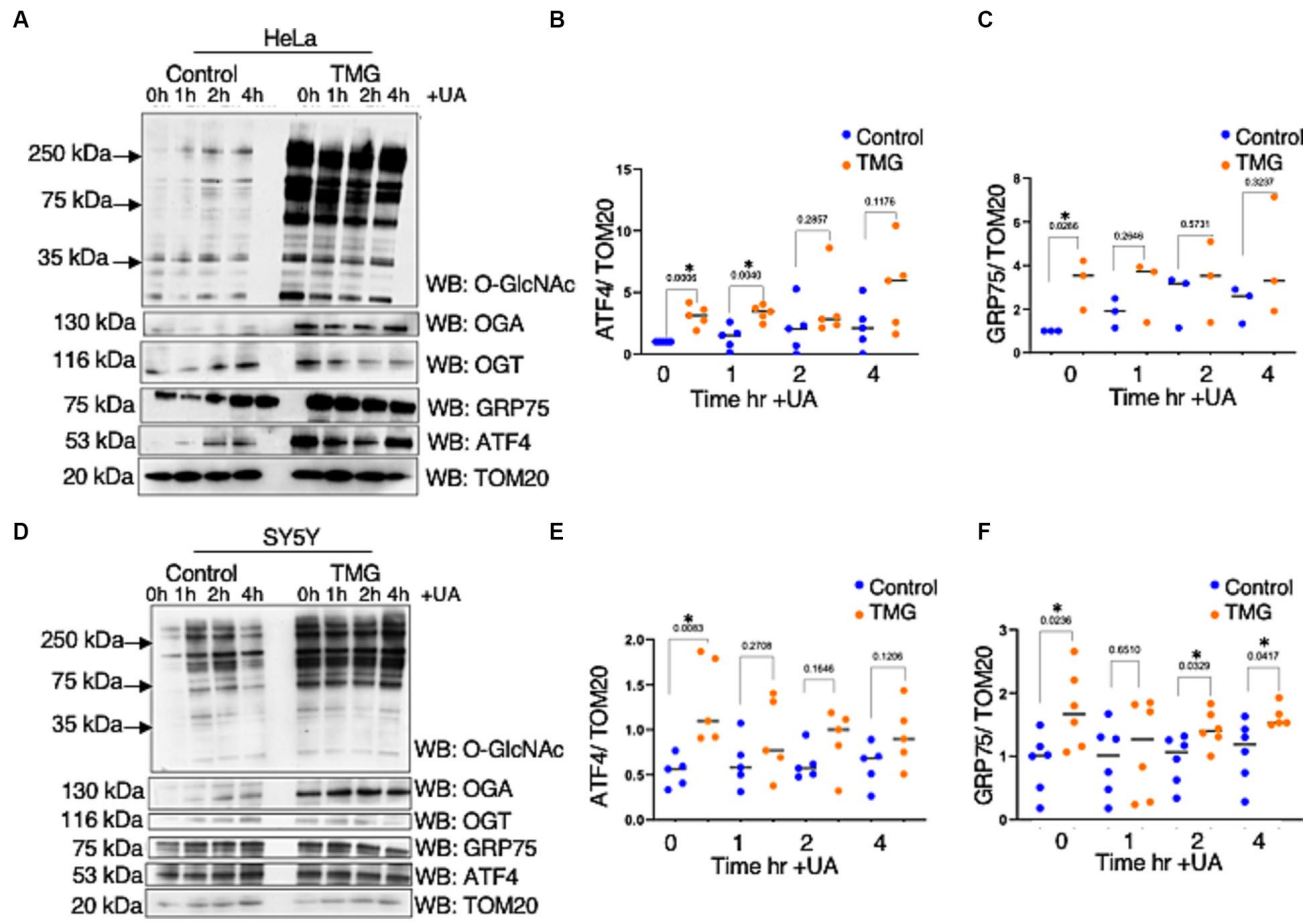
TMG elevates mitochondrial ATF4 and GRP75 in human cell lines. Western blot analysis of mitochondria isolated after stimulating mitochondrial stress using UA in HeLa cervical cancer cells **(A)** and SH-SY5Y neuroblastoma cells **(D)** subjected to long-term OGA inhibition with TMG. **(B,E)** Densitometry plot of ATF4 normalized to TOM20. **(C,F)** GRP75 normalized to TOM20. The dots represent the number of experimental trials. Experiments were performed with at least three biological replicates. Statistical significance was measured using an unpaired *t*-test analysis, and *p*-values are indicated on the plots. *p-values that are significant (*p* < 0.05).

As ATFS-1, the *C. elegans* ortholog of ATF4, can be imported into the mitochondrial matrix, we investigated whether TMG alters ATF4 levels in the mitochondria (Haynes et al., 2010; Lill et al., 2012). Under normal conditions, ATFS-1 is efficiently imported into the mitochondrial matrix and degraded. However, under mitochondrial stress, mitochondrial import is reduced, causing ATFS-1 to accumulate and traffic to the nucleus to activate the transcriptional response (Melber and Haynes, 2018). TMG significantly elevates the mitochondrial localization of ATF4 in HeLa and SY5Y (Figures 4A,B,D,E). Additionally, GRP75 is significantly elevated with higher O-GlcNAc (Figures 4A,C,D,F).

**Figure 5:**
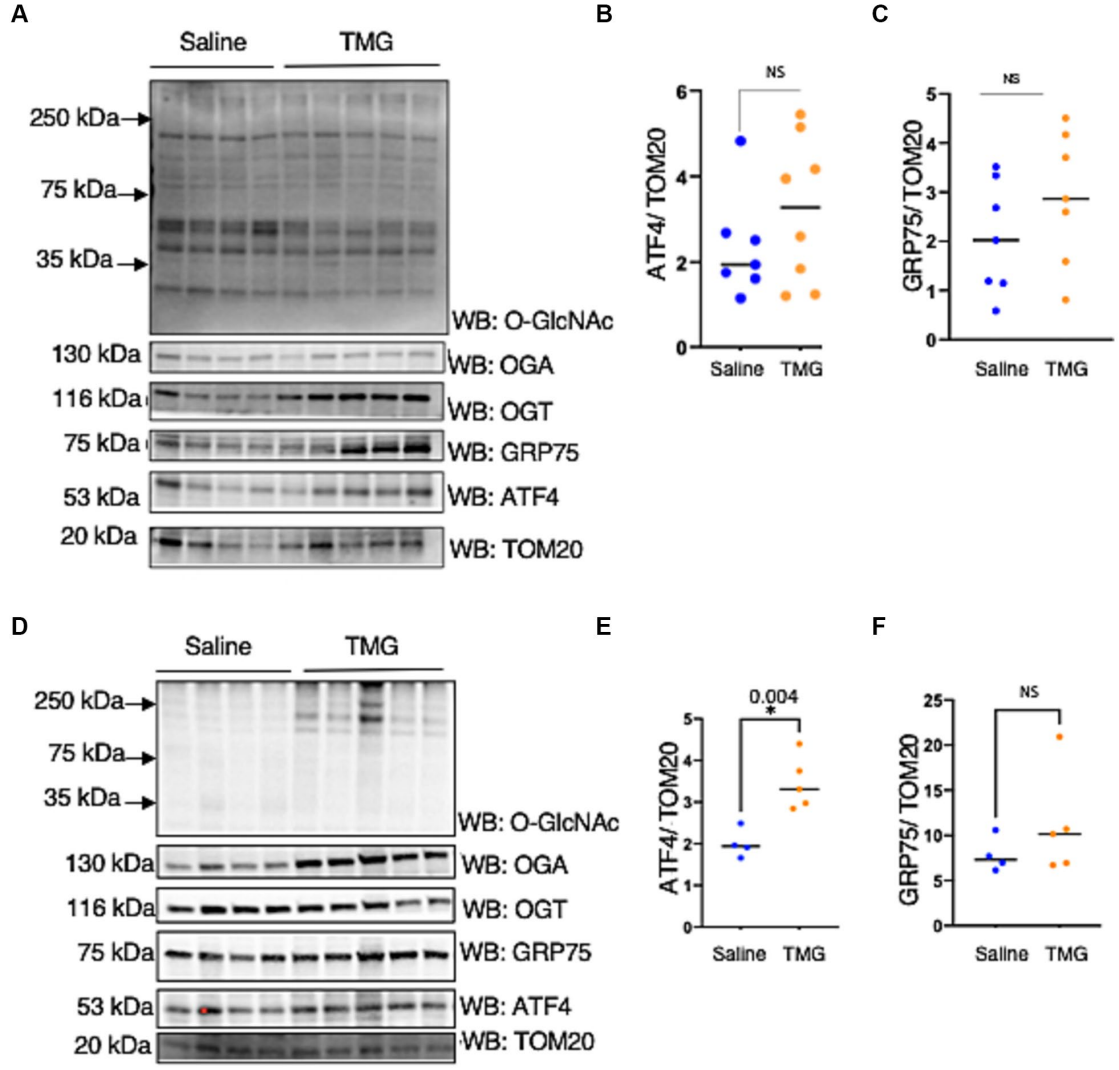
TMG increases mitochondrial ATF4 and GRP75 in isolated mice brains. Western blot analysis of brain mitochondria of WT-C57BL/6J male mice subjected to intraperitoneal TMG injections for 1 month **(A)** and 6 months **(D)**. The blots were probed for O-GlcNAc, OGT, OGA, TOM20, GRP75, and ATF4 **(A)**. **(B,E)** Densitometry plot of ATF4 normalized to TOM2. **(C,F)** GRP75 normalized to TOM20. The dots represent the number of mice. Experiments were performed with at least three biological replicates. Statistical significance was measured using an unpaired *t*-test analysis, and *p*-values are indicated on the plots. *p-values that are significant (*p* < 0.05).

Next, we investigated whether mitochondria isolated from the brains of C57BL/6 J mice intraperitoneally injected with TMG replicated the cell line data. There was a slight change in ATF4 and GRP75 in the mitochondria isolated from the brains of C57BL/6 J male and female after 1 month of TMG injections (Figures 5A–C). Next, we extended the injections for a period of 6 months in C57BL/6J mice to simulate a long-term inhibition of OGA. We found that ATF4 was significantly increased in males after 6 months of TMG injections, and GRP75 slightly increased compared with the control (Figures 5D–F).

**Figure 6:**
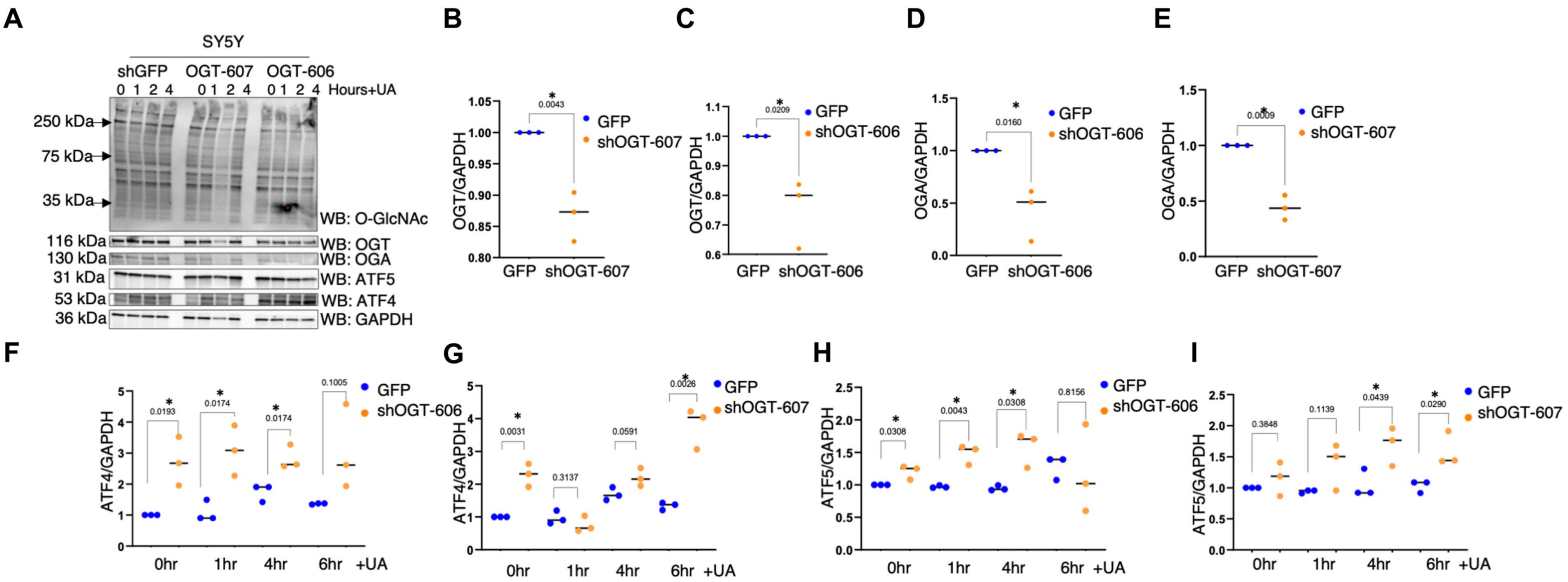
OGT-KD initiates the ISR in SH-SY5Y. Western blot analysis of samples harvested after stimulating the mitochondrial stress time course in SH-SY5Y OGT KD cells 606 and 607 **(A)**. **(B–D)** Densitometry plot of OGT and OGA normalized to GAPDH. **(F,G)** Densitometry plot of ATF4 normalized to GAPDH. **(H,I)** Densitometry plot of ATF5 normalized to GAPDH. OGT KD of 606 (*n* = 3) and 607 (*n* = 3) in which dots represent the number of experimental trials (n). Statistical significance was measured using a paired *t*-test analysis, and *p*-values are indicated on the plots. *p-values that are significant (*p* < 0.05).

**Figure 7:**
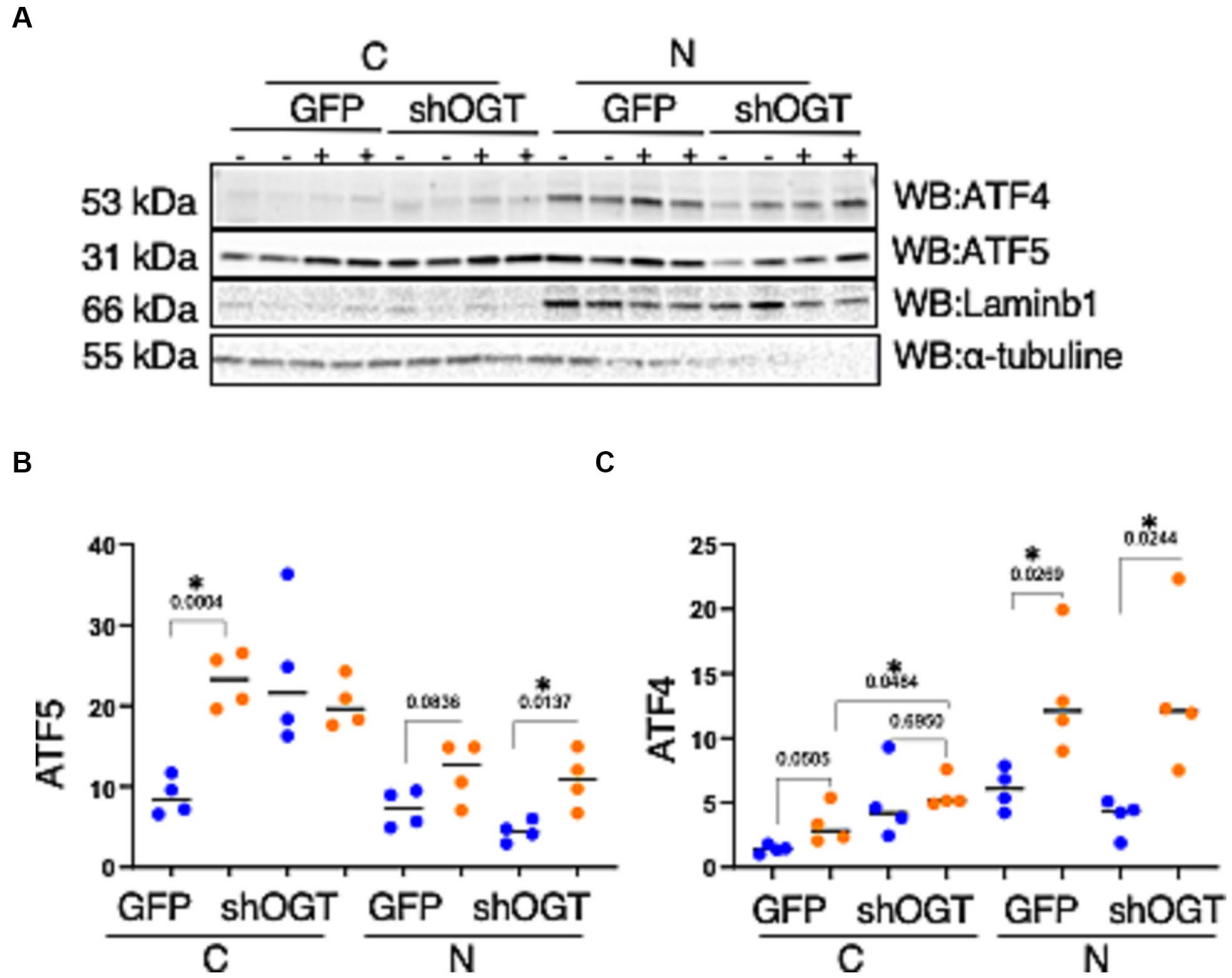
Mitochondrial stress elevates the transportation of ATF4 and ATF5 to the nucleus and OGT-KD does not impact transportation. Nuclear/cytoplasmic extraction was performed in SH-SY5Y OGT KD after stimulating mitochondrial stress with UA for 6 h. **(A)** Western blot analysis was performed for Laminb1 (a marker of the nuclear fraction), α-tubuline (a marker of the cytoplasm), ATF4, and ATF5. **(B)** Densitometry plot of ATF5 normalized to the appropriate loading control. **(C)** ATF4 normalized to the appropriate loading control. All experiments were performed with at least three biological replicates. Statistical significance was measured using an unpaired *t*-test analysis, and *p*-values are indicated on the plots. *p-values that are significant (*p* < 0.05). N, nuclear; C, cytoplasmic.

To understand how a disruption to O-GlcNAcylation affects ISR^mt^, we assessed ATF4 and ATF5 protein expression in OGT KD SH-SY5Y. We set up a similar mitochondrial stress stimulating time course with SH-SY5Y OGT KD 606 and 607 cells (two different OGT KD short-hairpin shRNA expressing cell lines). OGT KD caused O-GlcNAc, OGA, and OGT to decrease as expected (Figures 6A–E). Interestingly, OGT KD alone increased the protein expression of both ATF4 and ATF5 (Figures 6A,F–I). To examine whether stimulating mitochondrial stress increases the transportation of ATF4 to the nucleus, nuclear isolation was conducted 0-h and 6-h post UA addition. UA increased the cytoplasmic level of ATF4 and ATF5 compared with non-UA. Importantly, UA increased the transport of ATF4 and ATF5 to the nucleus (Figures 7A–C). UA caused an equal level of ATF4 and ATF5 transport to the nucleus in OGT KD and GFP (Figures 7B,C).

**Figure 8:**
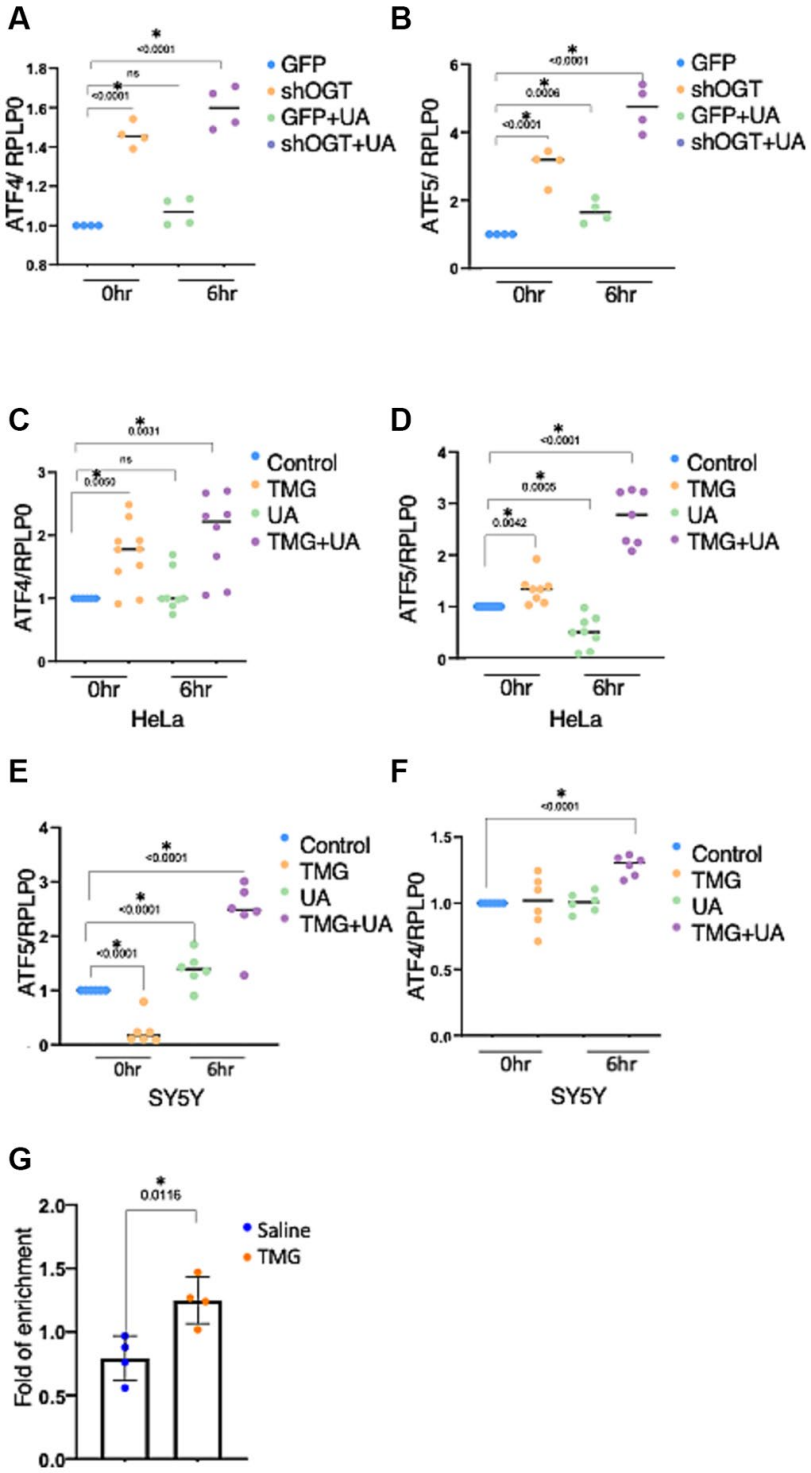
OGT KD increases the transcription of the stress response. The transcription levels of ATF4 and ATF5 were measured by RT-qPCR after stimulating mitochondrial stress with UA for 6 h. **(A–F)** Ribosomal protein lateral stalk subunit P0 (PRLP0) was used as an internal control. **(A,B)** SH-SY5Y OGT KD transcriptional level of ATF4 and ATF5 normalized to PRLP0. **(C,D)** HeLa TMG-treated transcriptional levels of ATF4 and ATF5 normalized to PRLP0. **(E,F)** SH-SY5Y TMG-treated transcriptional levels of ATF4 and ATF5 normalized to PRLP0. **(G)** A ChIP assay was performed using female mice brains treated with TMG for 1 month. ATF4 ChIP DNA was analyzed by qPCR using a set of primers targeting the ATF5 promoter. Normal rabbit IgG served as a negative control. All experiments were performed with at least three biological replicates. Statistical significance was measured using an unpaired *t*-test analysis, and *p*-values are indicated on the plots. *p-values that are significant (*p* < 0.05). ns, not significant.

OGT-KD significantly increased the mRNA expression of ATF4 and ATF5 (Figures 8A,B). UA-treated OGT-KD further elevated the mRNA levels of ATF4 and ATF5 (Figures 8A,B). Similarly, prolonged OGA inhibition increased the mRNA expression of ATF4 and ATF5, and UA further elevated them in HeLa (Figures 8C,D). However, prolonged OGA inhibition in SY5Y significantly decreased ATF5 mRNA expression (Figure 8E), which was consistent with lower ATF5 protein expression upon TMG treatment (Figures 2D,E). Interestingly, introducing mitochondrial stress to TMG-treated SY5Y increased the mRNA expression of ATF4 and ATF5, replicating the results with HeLa (Figures 8E,F).

Next, we questioned if O-GlcNAc alters the occupancy of ATF4 at its targeted gene promoters, ATF5. ATF4 binding site at the ATF5 promoter has been identified previously in mice (Teske et al., 2013). Therefore, we carried out chromatin immunoprecipitation (ChIP) analyses to measure ATF4 occupancy on ATF5 promoter sites from female mice TMG-treated brains. We found a significant increase in ATF4 binding to the ATF5 promoter in TMG treated mice brains (Figure 8G).

**Figure 9:**
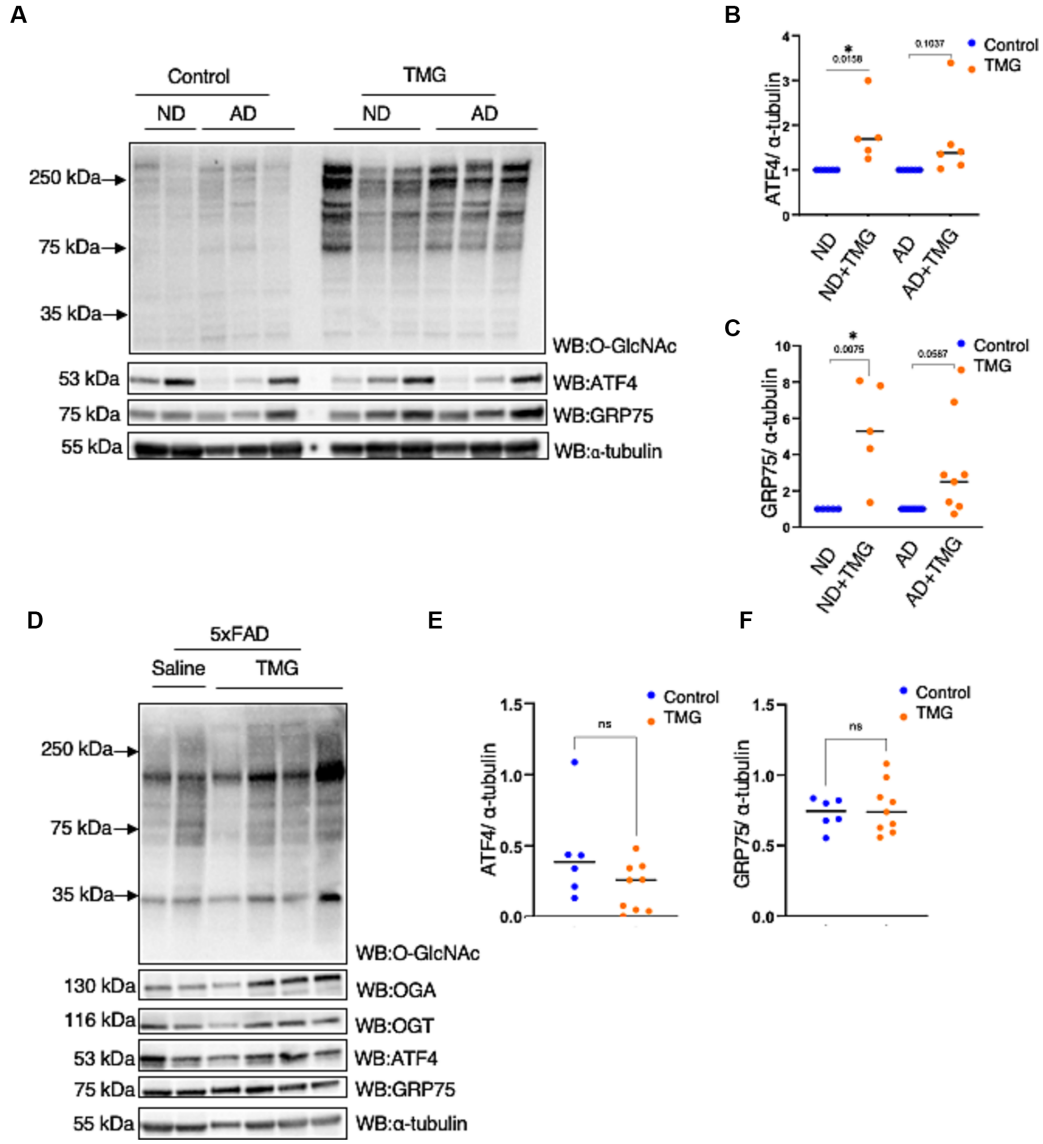
In AD, the ATF4 response to O-GlcNAc alteration is altered. **(A)** Differentiated organoids from AD patients and healthy individuals of both genders were treated with TMG for 2 weeks and lysed for western blotting. **(B)** Densitometry plot of ATF4 normalized to α-tubulin. **(C)** Densitometry plot of GRP75 normalized to α-tubuline. **(D)** Western blot analysis of the total brain lysate of 5 × FAD female and male mice subjected to intraperitoneal TMG injection for 1 month. **(E)** Densitometry plot of ATF4 normalized to α-tubuline. **(F)** Densitometry plot of GRP75 normalized to α-tubuline. All experiments were performed with at least three biological replicates. Statistical significance was measured using an unpaired *t*-test analysis, and *p*-values are indicated on the plots. *p-values that are significant (*p* < 0.05). ns, not significant.

Because ATF4 is found at high levels in AD without restoring mitochondrial function, we investigated whether ATF4 is responsive to O-GlcNAc alteration in a human *in vitro* AD model. We treated differentiated organoids from sex- and aged-matched AD patients and healthy individuals of both genders with TMG for 2 weeks. O-GlcNAc levels were significantly higher in the TMG-treated cells, as expected (Figure 9A). ATF4 and GRP75 were significantly elevated in prolonged TMG-treated organoids that were differentiated from normal individuals (Figures 9A–C). Interestingly, TMG slightly increased ATF4 and GRP75 expression in organoids derived from sporadic Alzheimer patients (Figures 9A–C). In a 5 AD-linked mutations mouse model (5 × FAD), there was no change in ATF4 and GRP75 after 1 month TMG treatment (Figures 9D–F).

## Discussion

**Figure 10:**
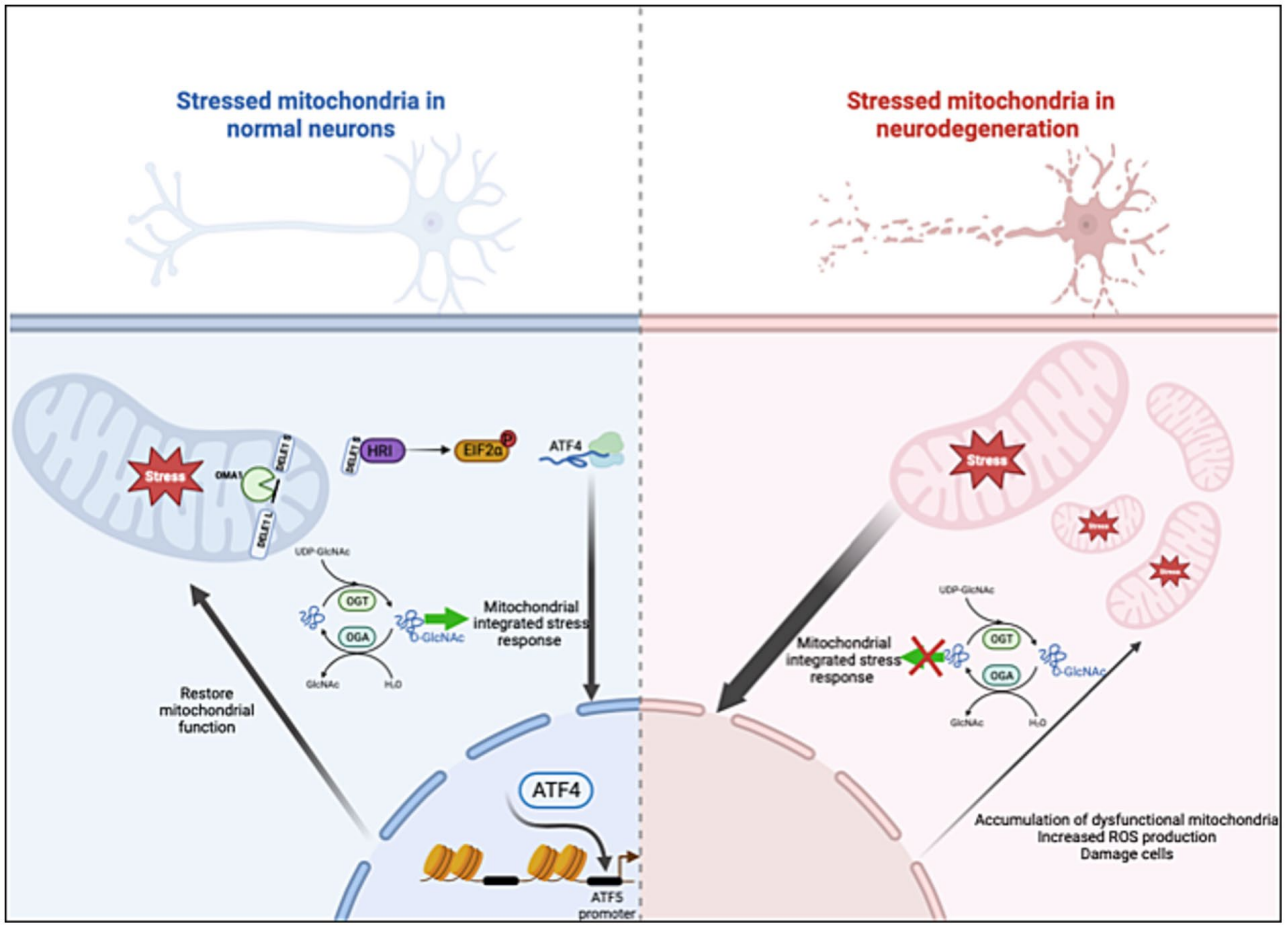
O-GlcNAcase inhibition stimulates the ISR^mt^ with a limited effect in AD. Mitochondrial dysfunction triggers the integrated stress response (ISR^mt^), in which the phosphorylation of eukaryotic translation initiation factor 2α results in the translation of ATF4. ATF4 activates gene expression to restore mitochondrial function. *O*-GlcNAcase inhibition elevates ATF4 and increases ATF4 occupancy at the ATF5 promoter site. However, the ISR^mt^ is consistently activated in AD without restoring mitochondrial homeostasis. Manipulating O-GlcNAc in AD does not impact the ISR^mt^. Created with BioRender.com.

In this paper, we demonstrate the fundamental role of O-GlcNAcylation in controlling ISR^mt^. Prolonged OGA inhibition elevates ATF4 and GRP75 protein expression in cell lines, mouse brains, and control human organoids. Disrupting O-GlcNAc by either OGT-KD or TMG treatment while inducing mitochondrial stress with UA elevates the mRNA and protein expression of genes targeted by the ISR^mt^ in SH-SY5Y. Mitochondrial ATF4 and GRP75 levels increase with higher O-GlcNAc levels in both cell lines and mouse brains. Moreover, chromatin immunoprecipitation reveals that O-GlcNAc directly regulates the ATF4 promoter by upregulating gene expression in mouse brains. Importantly, ATF4 response to O-GlcNAc alteration is impaired in both a mouse AD model and a human *in vitro* AD organoid model (Figure 10). These results indicate O-GlcNAc regulation of the mitochondrial retrograde stress response, but it has a limited effect in AD.

O-GlcNAc levels increase rapidly in response to diverse cellular stress responses such as heat shock (Zachara et al., 2011), hypoxia (Ngoh et al., 2009), and oxidative stress (Lee et al., 2016). O-GlcNAc modifications of most proteins are dynamic and reversible, returning to normal within 24–48 h after resolving the stress (Zachara et al., 2004). Alterations in the rate of O-GlcNAc cycling, the addition followed by the removal of the modification, alters cellular response to stress. Increases in O-GlcNAc levels mediate stress tolerance and survival. For example, heat shock increases O-GlcNAc levels and directly leads to elevating heat shock proteins (HSP) HSP70 and HSP40, producing a cytoprotective effect. Our study agrees with previous findings that increases in O-GlcNAc levels protect stressed mitochondria via elevating the ISR^mt^, which in turn increases mitochondrial chaperon GRP75. Mitochondrial chaperones have fundamental functions in regulating oxidative stress, chaperoning misfolded proteins and reducing neurotoxicity (Voos and Röttgers, 2002; Voloboueva et al., 2008; Xu et al., 2009; Lackie et al., 2017). Therefore, OGA inhibition could elevate mitochondrial homeostasis via elevating mitochondrial chaperone levels. In contrast, neuronal OGT-KD lowers O-GlcNAcylation, producing a high level of stress. In these cells, the activation of the ISR is significantly elevated. These data are consistent with previous studies in liver in which hepatocyte-specific OGT-KO mice generated reactive oxygen species, stimulating stress response signaling pathways such as NRF2 and antioxidant glutathione (Tan et al., 2017; McGreal et al., 2018). Surprisingly, the elevation of stress signaling in the loss of OGT was protective against acetaminophen-induced liver injury. Contradictory to the decrease in O-GlcNAc, treating mice with TMG exacerbated acetaminophen-induced hepatotoxicity via increasing the activation of c-Jun N-terminal kinases (McGreal et al., 2018). Thus, stimulated stress signaling in OGT-KO promotes cellular tolerance against severe stress, while increasing O-GlcNAc levels show the opposite. Collectively, these studies suggest that the manipulation of O-GlcNAc levels could provide a protective effect in a context-specific manner.

Increasing O-GlcNAc provides a cytoprotective effect through the elimination of protein aggregates via stimulating the ISR in *C. elegans*. Increasing UDP-GlcNAc levels via gain-of-function mutations in GFAT1 or supplementation with GlcNAc in *C. elegans* stimulates ISR, leading to the accumulation of ATF4 (Horn et al., 2020). Our data show similar results: increasing O-GlcNAcylation via TMG stimulates ISR in mammalian cell-lines and brain tissues. Previously, TMG treatment stimulated the autophagic clearing of toxic proteins without identifying the mechanism (Zhu et al., 2018; Horn et al., 2020). Our results suggest that O-GlcNAc mediated the ISR^mt^, which is one potential mechanism for initiating autophagy. Brains from TMG-treated mice showed high levels of ATF4, with higher ATF4 binding at the ATF5 promoter site initiating the adaptive stress response. ATF4 and ATF5 are required to maintain mitochondrial homeostasis by increasing the transcription of chaperones, proteases, and mitophagy genes (B’chir et al., 2013; Fiorese et al., 2016; Slavin et al., 2022; Figure 10). Thus, OGA inhibition could aid the brain in combatting the accumulation of dysfunctional mitochondria via stimulating ISR^mt^ in mitochondrial diseases.

Our OGT-KD data showed elevated levels of the ISR. However, previous studies have shown crucial roles of OGT in targeting two essential autophagy/mitophagy proteins: Unc-51-like-kinase 1 (ULK1) and PTEN-induced putative kinase 1 (PINK1) (Pyo et al., 2018; Murakami et al., 2021). ULK1 O-GlcNAcylation is essential for phagophore formation and the initiation of autophagy. OGT regulates PINK1 at the transcriptional level and OGT-deficient cells show a decreased PINK1 level with accumulating defective mitochondria. The elevated levels of the ISR in OGT-KD could be a cause, an effect, or both for failing to restore mitochondrial quality control: (1) disruption in O-GlcNAc homeostasis due to the loss of OGT is elevating the stress, or (2) the loss of OGT impairs the cellular quality control mechanism, elevating cellular stress. In both scenarios, OGT is essential for maintaining cellular homeostasis.

OGA inhibitors are now in Phase I or II clinical trials as a treatment for AD (Selnick et al., 2019; Shcherbinin et al., 2020; Permanne et al., 2022). Several studies suggest that activating the ISR^mt^ provides neuroprotection, specifically improving mitochondrial function (Zhang et al., 2020; Wang et al., 2021). However, there are limited studies investigating the protective effect of OGA inhibitors in the context of the ISR. We showed that TMG elevates the ISR in WT mice brains and human organoids. However, our data show that, in neurodegenerative models, increasing O-GlcNAc levels can further activate the ISR. Because of the constant activation of the ISR^mt^ in AD (Abisambra et al., 2013; Ma et al., 2013; Costa-Mattioli and Walter, 2020), the stress signaling is likely maximized, blocking the effect of TMG (Figure 10). ATF4 is a master regulator of an essential set of genes regulating cellular homeostasis. Targeting OGA might not alter ISR^mt^ expression in AD but ATF4 activity could be altered. Therefore, understanding the mechanism of how O-GlcNAc modulates ATF4 activity in normal vs. disease conditions is essential for providing insights into how to restore mitochondrial function in AD. Future studies are required to understand the effect of OGA inhibition on ATF4 targeted gene expression programs in the context of AD to answer whether OGA inhibition is initiating pro-survival signaling through ATF4 regulation. Answering these questions will provide a novel mechanism for neurodegenerative targeting therapy.

## Data availability statement

The original contributions presented in the study are included in the article/Supplementary material, further inquiries can be directed to the corresponding author.

## Ethics statement

The animal study was approved by the University of Kansas Medical Center Animal Care and Use Committee approved all experiments in this study. The study was conducted in accordance with the local legislation and institutional requirements.

## Author contributions

IA: Conceptualization, Data curation, Investigation, Methodology, Writing – original draft. MC: Investigation, Writing – original draft. HW: Investigation, Methodology, Resources, Writing – review & editing. SE: Investigation, Writing – review & editing. AQ: Investigation, Writing – review & editing. WD: Investigation, Methodology, Project administration, Writing – review & editing. HF: Investigation, Writing – review & editing. AD: Investigation, Writing – review & editing. RS: Conceptualization, Resources, Writing – review & editing. CS: Conceptualization, Funding acquisition, Resources, Writing – review & editing.
